# What Can the Brain Teach Us about Winemaking? An fMRI Study of Alcohol Level Preferences

**DOI:** 10.1371/journal.pone.0119220

**Published:** 2015-03-18

**Authors:** Ram Frost, Ileana Quiñones, Maria Veldhuizen, Jose-Iñaki Alava, Dana Small, Manuel Carreiras

**Affiliations:** 1 The Hebrew University, Jerusalem, Israel; 2 BCBL, Basque center of Cognition, Brain and Language, Donostia-San Sebastian, Spain; 3 Haskins Laboratories, New Haven, United States of America; 4 The John B. Pierce Laboratory, New Haven, United States of America; 5 Basque Culinary Center, Donostia-San Sebastian, Spain; 6 Yale Medical School, New Haven, United States of America; 7 University of Cologne, Köln, Germany; 8 IKERBASQUE, Basque Foundation for Science, Bilbao, Spain; 9 Department of Basque Language and Communication, University of the Basque Country EHU/UPV, Bilbao, Spain; Barnard College, Columbia University, UNITED STATES

## Abstract

Over the last few decades, wine makers have been producing wines with a higher alcohol content, assuming that they are more appreciated by consumers. To test this hypothesis, we used functional magnetic imaging to compare reactions of human subjects to different types of wine, focusing on brain regions critical for flavor processing and food reward. Participants were presented with carefully matched pairs of high- and low-alcohol content red wines, without informing them of any of the wine attributes. Contrary to expectation, significantly greater activation was found for low-alcohol than for high-alcohol content wines in brain regions that are sensitive to taste intensity, including the insula as well as the cerebellum. Wines were closely matched for all physical attributes except for alcohol content, thus we interpret the preferential response to the low-alcohol content wines as arising from top-down modulation due to the low alcohol content wines inducing greater attentional exploration of aromas and flavours. The findings raise intriguing possibilities for objectively testing hypotheses regarding methods of producing a highly complex product such as wine.

## Introduction

Today’s globalization offers wine consumers an impressive variety of wines coming from almost anywhere in the world. Their eventual choices reflect a series of criteria, such as price, origin, vintage, previous exposure, prestige, or even label design and bottle shape (e.g., [1, 2]). However, inevitably, their choices also reflect gustatory preferences. The red ruby or yellow golden liquid in the glass has an amazing array of potential aromas and flavors. These emerge to some extent from the “terroir” (i.e., climate and soil) determinants of a wine region, so that similar grape varieties express dramatically different characteristics. However, aromas and flavors of wine are to a large extent determined by the producer’s specific style of winemaking.

Winemaking styles reflect historical traditions. Overall California’s winemaking style is more similar to that of Australia or Chile (often labeled “new-world wines”), but different from that of France, Italy, or Spain (often labeled “old-world wines”). However, even within a given narrow wine region, there is a huge variance in styles of winemaking, that are characterized by a series of individual decisions taken by the winemaker throughout the year. These concern, for example, reduction of crop per acre, date of harvest, chaptalization, filtering, microoxygenation, temperature of fermentation, to name just a few. How do individual winemakers choose which way to go? Setting aside obvious factors such as tradition or ideologies, to a large extent, winemakers’ decisions are often based on their beliefs regarding the gustatory preferences of consumers. Very little systematic well-controlled experimental research, however, has been conducted to validate these underlying assumptions. To our knowledge, the present paper is the first study that examines brain signature to a specific wine component.

Our initial investigation focuses on alcohol level. We selected alcohol level, because it is a critical determinant manipulated during winemaking, and it has seen significant changes in recent years. In the last two or three decades, there is a remarkable trend towards producing “powerful wines”, having higher levels of alcohol. Whereas 30 years ago levels of alcohol of 12% or 12.5% were common, today’s bottles on the shelves commonly display levels of 14%, 14.5% or even 15%. Alcohol content is determined by the amount of sugar in the grape. In the presence of yeast, the sugar ferments into alcohol, so that higher sugar contents result in higher alcohol contents. To some extent, today’s higher alcohol levels are related to overall global warming (warm climate results in higher sugar content). However, sugar content can also be manipulated by winemakers. This is done mainly through chaptalization (a process of adding sugar to the unfermented crushed grapes, a practice that is prohibited in some wine regions), or through later harvest, so that the water content within the grape would be reduced, thereby increasing its ratio of sugar to water.

Why the trend? One can offer various hypotheses and speculations related to stylistic preferences of influential wine critics or simply to social fashion. Higher alcohol content is commonly associated with “powerful”, “intense”, full-bodied” wine. Note that this approach to winemaking is not unanimously accepted, and some wine experts argue to the contrary, mainly, that high-alcohol content masks the subtle aromas and flavor of wine. Nevertheless, many wine producers (at least in “new world styles”) seem to follow this trend, probably assuming that such wines are, on the average, more appreciated by wine consumers. This hidden common assumption has never been systematically investigated empirically. This is the aim of the present pioneering investigation.

The two chemical senses, taste and smell, are much less quantifiable than other human senses such as visual, auditory, or somatosensory perception. Participants’ overt assessments have typically a very large variance, are often unreliable, and highly influenced by expectations [2, 3]. We therefore, chose to use functional magnetic imaging to record covert brain reactions to high- and low- alcohol content wines in regions of the brain critical for flavor processing and food reward, without informing the subject of any of the wine attributes.

The main methodological concern with such an experimental approach is that differential responses, if indeed found, could be related not to the manipulated dependent variable (high-or low-alcohol content), but perhaps to another wine attribute. We addressed this problem in several ways. First, the low- and high-alcohol content wines were selected and matched given a series of parameters that are known to affect aroma and flavor. These include: wine region (all wines in the study were red Spanish coming from Rioja, Navarra, and Cataluña), grape variety, vintage, and overall quality in term of marketing price. More importantly, we matched the high- and low-alcohol content wines also on exact objective measures of pH and levels of residual sugar. Second, we selected four independently matched pairs of high- vs. low-alcohol contents wines, so that each participant was tested with one pair of wines considered as a random variable. Finally, at the end of the experiment, participants conveyed their overt preferences to the two wines they have consumed during the experiment. To preview our findings, our procedure of matching the wines indeed resulted in that subjective rankings of the high- and low-alcohol content wines outside the scanner were virtually identical. Nevertheless, significant differential activation was found for the two types of wines in the right insula and the cerebellum, brain regions implicated in taste and flavor processing [4].

## Materials and Methods

### Participants

Twenty-six healthy relatively inexperienced wine consumers participated as paid volunteers in the study. Wine consumption habits were assessed through a questionnaire (S1 Appendix), and only those subjects that consume wine on a regular basis, but not more than once per week, were selected for this experiment. Participants had no psychiatric or neurological disorders, or smell, taste, or digestive impairments. All participants gave their written informed consent in accordance with guidelines approved by the Ethics and Research Committees of the Basque Center on Cognition, Brain and Language (BCBL). The Ethics Committee of the BCBL has specifically approved this study. The quality of the fMRI data of each individual subject was explored using the Artifact Repair toolbox (Gabrieli Cognitive NeuroScience Lab; http://cibsr.stanford.edu/tools/ArtRepair/ArtRepair.htm). From this analysis three participants whose fMRI data exhibited more than 40% of the scan-to-scan motion estimation higher than 1 mm, were excluded from following statistical analysis. In addition, two participants were also eliminated because they reported in the post-scanner test that they felt discomfort with the wines and/or during the experimental procedure. A total of twenty-one participants (eight females), with ages ranging from 22 to 42 years (mean = 28.6, standard deviation = 4.8), were used to estimate the group effects.

### Stimuli and experimental design

**Fig 1:**
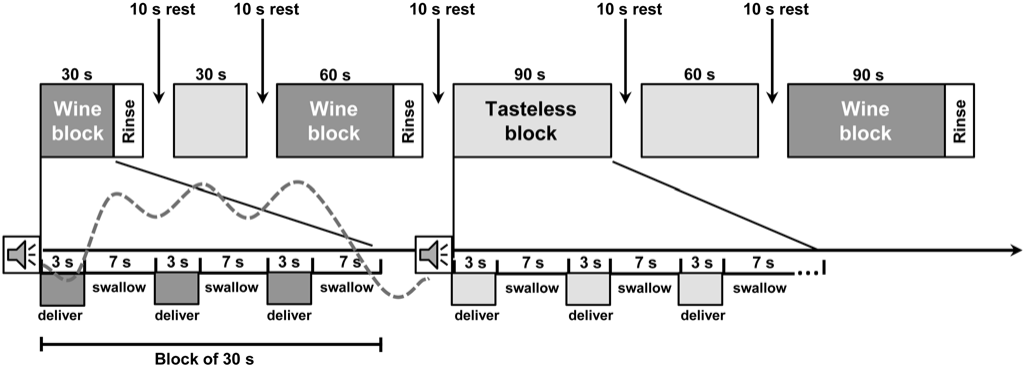
Each scan consists of a serial delivery of one of three different types of taste stimuli (a block). A block consists of a series of 3, 6 or 9 presentations. An auditory cue was presented before each block to alert participants which type of stimuli (wine or solution) will be delivered. Each presentation starts with a 0.75 ml delivery of liquid over 3 s followed by 7 s in which to swallow. Each wine block is followed by a rinse (0.75 ml distilled water). Before the start of a new block there is a rest period of 10 seconds. Blocks vary in length between 30 and 90 seconds, the order of blocks is counterbalanced across subjects. The hemodynamic response function predicted for each block was schematically represented with the grey dotted line.

Each subject participated in four consecutive sessions consisting of four randomized repetitions of a block design functional scan (see Fig. 1). Each scan consisted of a serial delivery of three different types of taste stimuli (Wine Low [Low level of alcohol], Wine High [High level of alcohol] and tasteless solution). An auditory cue was presented before each tasting period to alert participants about which type of stimuli (wine or solution) will be delivered. In order to optimize the design statistical efficiency, the duration of the taste period was randomized across blocks (30.0, 60.0 and 90.0 seconds, in the proportion of 3:3:3). After each wine block, distilled water was delivered in order to rinse the mouth and prepare participants for the next block. After that, a baseline rest period was presented for ten seconds.

All the wines used were red, and the difference between the levels of alcohol of the two wines was about 1.5% (Low alcohol contents: between 13 and 13.5%; High alcohol contents: between 14.5 and 15.0%). The PH of the two types of wines were virtually identical (PH = 3.68, and 3.69, for Low- and high-alcohol content wines respectively). Levels of residual sugar were very low as for most dry red wines, (2.4 and 1.8 g/l, that is, 0.24% and 0.18%, for Low- and high-alcohol content respectively). The tasteless solution consisted of 12.5 mM KCl and 1.25 mM NaHCO_3_ in distilled water. Each subject was tested with only one pair of wine; however, as specified above we used four different pairs of wines counterbalanced across participants, so that our findings would not depend on a specific contrast of two wines. Solutions and wines were delivered through a gustometer system as 0.75 ml per trial, ~ 50 ml per run and ~200 ml in total. Following scanning, participants were given two glasses of the wine, containing the wines with which they were tested, in a counterbalanced order, and rated their liking of the two wines on a 1 to 20 scale. The low- and high-content alcohol wines ratings were virtually identical at about 10.5 on the scale.

The gustometer system consisted of 11 programmable BS-8000 syringe pumps (Braintree Scientific, Braintree, MA, USA) connected to a computer. This computer was synchronized with the scanner through a parallel port. Each pump controlled a 60 ml syringe connected to a 15 foot length of Tygon beverage tubing (Saint-Gobain Performance Plastics, Akron, OH, USA) that was passed through the waveguide to the magnet room. These tubes were bolted to the head-coil using a plastic piece that allowed delivery of the liquids into the subjects’ mouths. This system has been consistently used in previous neuroimaging studies [4, 5, 3].

### Image acquisition

Scanning was carried out on a Siemens MAGNETOM Trio, A Tim System 3-T scanner, using a standard 32 channel phased-array surface coil (Siemens, Erlangen, Germany), which provided high spatial resolution and signal-to-noise ratio. In all subjects BOLD-contrast-weighted echoplanar images for functional event-related scans consisted of 46 axial slices of 2.5 mm thickness (with 2.5 mm between slices) that covered the whole brain. In-plane resolution was 2.5 x 2.5 mm, with the following parameters: field of view (FOV) = 1342 x 1343 mm; matrix = 76 x 76; echo time (TE) = 30 ms; repetition time (TR) = 3s with no time gap; flip angle = 90°. The first volumes of each run were discarded to allow for T1 equilibration effects. Subsequently, a MPRAGE T1-weighted structural image (1 x 1 x 1 mm resolution) was acquired with the following parameters: TE = 2.97 ms, TR = 2530 ms, flip angle = 7° and FOV = 256 x 256 x 160 mm^3^. This yielded 176 contiguous 1 mm thick slices.

### Functional data analysis

Functional data were analyzed using SPM8 and related toolboxes (http://www.fil.ion.ucl.ac.uk/spm). Raw functional scans were slice-time corrected taking the middle slice as reference, spatially realigned, unwarped, coregistered with the anatomical T1 [6] and normalized to the MNI space using the unified normalization segmentation procedure. Global effects were then removed using a voxel-level linear model of the global signal proposed by Macey and colleagues [7]. Detrending fMRI time series were then smoothed using an isotropic 8mm Gaussian kernel. Resulting time series from each voxel were high-pass filtered (128s cut-off period).

Statistical parametric maps were generated by modeling univariate general linear model, using for each stimulus type a regressor obtained by convolving the canonical hemodynamic response function with delta functions at block onsets, with a duration corresponding to the length of each block. Rinses were included as a nuisance regressor with a duration of 0. We also included the six motion-correction parameters as nuisance regressors. Parameters of the GLM were estimated with a robust regression using weighted-least-squares that also corrected for temporal autocorrelation in the data (Diedrichsen & Shadmehr; http://www.bangor.ac.uk/~pss412/imaging/robustWLS.html). A pair-wise contrast comparing activity to *Wine Low + Wine High* relative to the *Tasteless* condition was performed. Resulting contrast images were then entered into a second level design analysis to enable population inferences. Only those clusters with a p-value corrected for multiple comparisons (FEW, p<0.05) were considered as significant and reported in the tables of results. All local maxima were reported as MNI coordinates [8]. To determine whether the regions resulting from the contrast *Wine Low + Wine High* > *Tasteless* distinguish between the two wines we performed a Region of Interest (ROI) analysis. The ROIs used were built in MNI space combining a functional and an anatomical criteria such that all voxels: a) were included in the group-level effect of *Wine Low + Wine High* > *Tasteless*; b) were connected to a local t maxima; and c) were included in one AAL structural ROI (http://marsbar.sourceforge.net/download.html#aal-structural-rois). Using this algorithm 30 ROIs were defined: 14 in the left hemisphere and 16 in the right hemisphere. Afterwards, for each ROI the contrasts *Wine Low* > *Wine High* and *Wine High* > *Wine Low* were computed.

## Results and Discussion

### 1. All Wines vs. Tasteless

**Fig 2:**
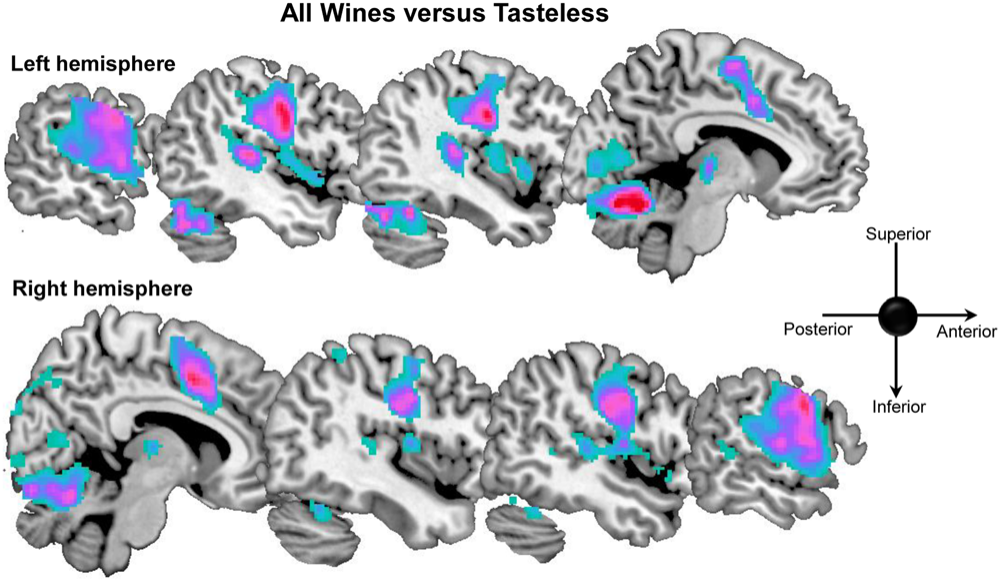
Sagittal view of brain activation for the contrast all wines vs. tasteless.

To characterize the functional neuroanatomical network involved in the processing of wine, we performed a pair-wise contrast comparing activity to Wine Low + Wine High relative to the Tasteless condition. This contrast showed increase of activation in several areas of the right hemisphere (rolandic operculum, post-central gyrus, cingulate gyrus, thalamus, inferior parietal lobule, lingual gyrus, fusiform and cerebellum) and on the left hemisphere (post-central gyrus, thalamus, supplementary motor area, superior temporal gyrus, fusiform and cerebellum 6) (see Table 1 and Fig. 2).

**Table 1:** All Wines versus Tasteless.

Hemisp	Region	No. voxels		T		Z		x,y,z {mm}
**Right**	Rolandic_Oper	4489		9.01		**5.56**		54 -2 14
	Postcentral			8.83		**5.5**		62 0 30
	Cingulum_Mid	2111		9.31		**5.64**		8 6 44
	Thalamus	388		5.87		**4.38**		14 -16 4
	Parietal_Inf	91	_	4.3	_	3.55	_	38 -44 56
	Lingual	2668		7.15		**4.92**		4 -80 -10
	Fusiform			6.01		**4.45**		24 -72 -14
	Cerebellum 6	8924		13.59		**6.64**		24 -64 -22
**Left**	Postcentral	4878		9.69		**5.75**		-44 -10 34
	Thalamus	388		6.89		**4.82**		-12 -20 4
	Supp_Motor_Area	2111		10.06		**5.85**		-2 2 52
	Temporal_Sup	8924		8.58		**5.42**		-44 -30 10
	Temporal_Sup			7.91		**5.2**		-64 -26 12
	Fusiform			6.85		**4.8**		-38 -58 -20
	Cerebellum 6	8924		16.35		**7.11**		-12 -64 -18

x, y, z {mm} = Coordinates in MNI space of local maxima. Z = Z scores. T = T scores. No. voxels = Number of voxels significantly activated inside the cluster belonging to each local maximum. Z scores are reported in bold if they are significant at the peak level after FWE or FDR correction (p<.05), if indicated by underline they are significant at p<.001 uncorrected. Inf: Inferior; ; Sup: Superior; Mid: Middle; Oper: Opercularis.

The cingulate cortex, post-central gyrus, rolandic operculum, ventral posterior medial (VPM) thalamus and cerebellum have been previously implicated in taste and flavour processing [9, 4]. The post-central gyrus and rolandic operculum correspond to taste cortex [10, 11, 4] and the gustatory nucleus of the thalamus is located in the VPM region [12]. Response to taste stimulation in the cerebellum correlates positively with intensity perception, which is thought to be represented in the cerebellum to optimize sensory-motor co-ordination during eating and drinking [4, 13]. The cingulate cortex also frequently responds to taste stimulation and plays a pivotal role in selective attention to taste, linking frontal and parietal attention regions with the insula and opercular taste cortex [14, 15]. The role of the superior temporal gyrus in taste processing is less explored but the region is activated by taste stimuli [10, 9] and has been suggested to be part of a distributed network that includes insula and thalamus, responsible for taste semantics [16].

### 2. Distinguishing between low- and high- alcohol wines

This contrast was the target of our study. After identifying the regions showing significantly different BOLD responses for *Wine Low + Wine High* relative to the *Tasteless* condition, we defined ROIs according the criteria described before (see Table 2). Subsequently, we compared in these ROIs the effects of degree of alcohol of the wines by contrasting in a pairwise manner activation responses to *Wine High > Wine Low* and *Wine Low > Wine High*.

**Table 2:** ROIs used for the region of interest analysis.

Right Regions	Right Hemisphere	Left Hemisphere
	x,y,z {mm}	x,y,z {mm}
Precentral	51 -2 35	-48 -3 35
Thalamus	10 -16 5	-11 -17 6
Insula	40 -6 8	-37 2 5
Anterior Insula	n.s.	-39 7 2
Posterior Insula	n.s.	-37 -7 9
Rolandic Operculum	55 -4 11	-50 -6 10
Anterior Cingulate	4 15 26	-2 14 28
Medial Cingulate	6 10 37	-3 9 37
Supplementary Motor Area	7 3 55	-4 0 56
Inferior Parietal	38 -43 53	n.s.
Superior Parietal	41 -44 57	n.s.
Superior Temporal Pole	58 7 -3	-53 8 -4
Superior Temporal	60 -18 6	-54 -22 8
Middle Temporal	66 -35 1	-55 -23 2
Cerebellum 6	22 -66 -21	-4 -66 -8
Cerebellum Crus 1	38 -62 -38	-30 -62 -38
Cerebellum 4 -5	10 -42 -4	n.s.
Cerebellum Crus 10	20 -38 -42	n.s.

x, y, z {mm} = Coordinates in MNI space of the center of mass of each ROI. All the regions included in this table emerge as significant at the peak level after FWE or FDR correction (p<.05) from the contrast All Wines vs. Tasteless. If “n.s.” (non significant) appears rather than the coordinates, then the corresponding region did not result in significant activation in the respective hemisphere.

**Fig 3:**
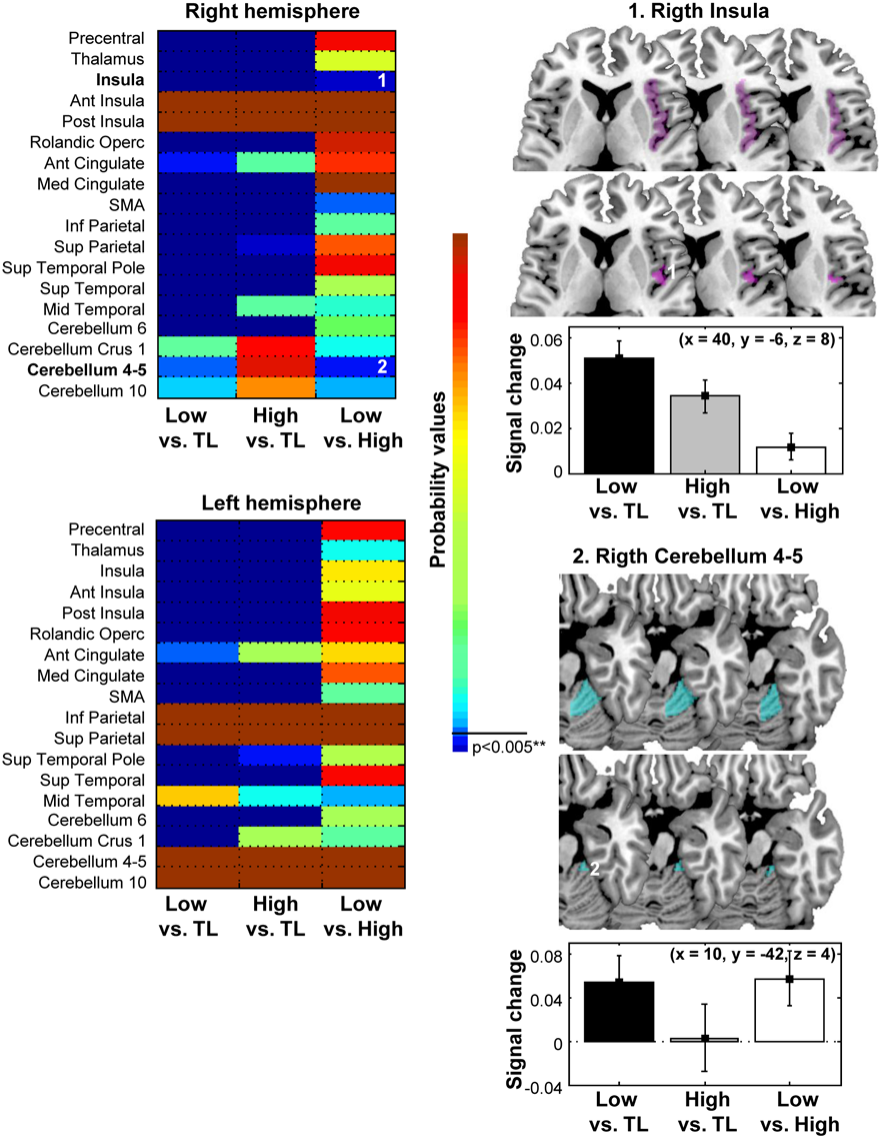
Right part: Axial view of brain activation for the contrast Low vs High degree of alcohol in the insula and the cerebellum. Right below each axial view corresponding charts of BOLD signal change for the contrasts Low vs. Tasteless, High vs. Tasteless, and Low vs. High in the insula and in the cerebellum. Left part: Rendering of probability values for the ROIs (right and left hemisphere) in the contrasts Low vs. Tasteless, High vs. Tasteless, and Low vs. High. Note that only the right insula and the right cerebellum (marked with 1 and 2) passed the corrected threshold for the Low vs. High contrast.

No effects were found for the comparison *Wine High > Wine Low*. In contrast, the *Wine Low > Wine High* comparison showed significant differences in the right insula and the cerebellum (see Fig. 3). Notably, these responses occurred in precisely the regions of insula and cerebellum previously shown to be sensitive to taste intensity perception (e.g., [4]). These studies have shown that BOLD response in the insula increase monotonically with intensity perception. This suggests that contrary to the common intuition regarding high-alcohol content wines (and thus contrary to the expected prediction), at least in our study, these wines induce weaker activation relative to the low-alcohol content ones. Given the large number of trials, we examined whether the pattern of results holds throughout the four sessions of the experiment. As can be seen in S1 Fig., similar results are indeed revealed across the four sessions.

Why might this be? Since in the current study the wines were closely matched for all physical attributes except for alcohol content it is unlikely that afferent sensory information is driving the stronger response to the low alcohol wines. In fact, stronger afferent signals should be generated to the higher alcohol wine, which should in turn produce the opposite pattern of results. Rather, a more likely possibility is that the increased activation reflects cognitive modulation of oral sensory perception. Insular responses to taste are strongly influenced by top-down modulation during attentional orienting [13, 3]. Relatedly, the cerebellum receives inputs from all sensory modalities and is thought to play a critical role in coordinating the acquisition of sensory information [17, 13, 18]. For example, in olfaction, the cerebellum coordinates sniff volume in relation to odor concentration [19]. A similar role for this circuit has been proposed for taste [4]. Our finding thus seems to suggest that the low-alcohol content wines induced a greater attentional orienting and exploration of the sensory attributes of wines relatively to high-alcohol content wines. We should also note that the right hemisphere taste cortex has been shown to respond to pleasant tastes compared with unpleasant or neutral ones [4, 20].

These findings then lead us to the ongoing debate among winemakers regarding the merits and drawbacks of the recent trend of producing high-alcohol content wines. The main criticisms of this “new world” approach to winemaking are that these wines often lack finesse, and also that *the high-alcoholic content overshadows the subtle flavours and aromas that the wine could exude*. Our findings regarding the stronger activation in the cerebellum for low-alcohol content wine seem to support the intuition of some professional wine experts that such lower-alcohol content wines have a better chance to induce greater sensitivity to the overall flavour expressed by the wine. Especially striking then is the fact that these differences were found for wine consumers that were not professional or experts.

In contrast to the findings of Plassmann and his colleagues [2], we did not find any differences in activation of the orbitofrontal cortex for the two types of wine. The target of the study by Plassmann et al. was to monitor participants’ reported pleasantness for wines that were falsely labelled as expensive or cheap. Activation in the orbitofrontal cortex has been shown to be correlated with self-reported pleasure (e.g., [21]). Note, however, that in contrast to Plassmann et al., the wines selected for our study were matched from the outset to produce *equal pleasantness*, and the subjective ratings of our participants of the high-and low-alcohol content wines were indeed virtually identical. Indeed, to test whether the difference between low- and high- alcohol wines could be explained by the subjective ratings, we estimated the correlation between the post-scanner ratings and the differential response pattern [Wine Low—Wine High] in those regions exhibiting significant difference between low- and high-alcohol wines. No significant results emerge from this analysis (r = 0.0, r = -0.19, p<0.4, for the cerebellum and right insula, respectively). Our results then seem to demonstrate that independent of overt self-reported pleasure, fine distinctions regarding wine attributes can still be detected using imaging. This raises the possibility that one could use fMRI to examine the neural basis of flavor preferences for complex products like wine, which elicit a diverse array of chemosensory and tactile sensations. Our findings, thus, point to possible directions for future research. First, we should note that similar to alcohol content, winemaking also involves controlling levels of acidity, amount of polyphenolic compounds (how tannic the wine would be), or extent of residual sugar (mainly in white wines). The complex interactions of these factors in terms of gustatory preferences could be subject to empirical investigation. Second, we should note that our present findings concern participants that mostly consume Spanish wines, often characterized by specific grape varieties, as well as climate and soil characteristics. The question whether the present reported preference to low-alcohol content wines would be generalized to other wine-producing areas, where wine consumers are exposed to different wine styles, still requires additional investigation.

## Supporting Information

S1 Appendix Wine Experience Questionnaire. (DOCX)

S1 Fig Sagittal view of brain activation for the contrasts Low vs. Tasteless (A) and High vs. Tasteless (B) for two different statistical models. The first model with the two first sessions is represented in the upper parts A and B. The second model with the first three sessions is represented in the lower parts A and B. Note that the results are similar for 2 and 3 sessions and for the whole 4 sessions as well (See Fig. 2 and Fig. 3).
(TIF)
