# Supplementary material for: What Can the Brain Teach Us about Winemaking? An fMRI Study of Alcohol Level Preferences
Source: PLoS One. 2015 Mar 18;10(3):e0119220. doi: 10.1371/journal.pone.0119220 (PMC4364721; doi:10.1371/journal.pone.0119220)
Supplement: S1 Appendix — (DOCX) [file pone.0119220.s001.docx]

**Appendix 1 (Wine Experience Questionnaire)**

1. Do you consider yourself an expert on wine? (circle answer)

Yes No

2. How often do you drink wine? (circle answer)

once a year once a month twice a month

once a week a few times per week every day

3. How much do you like drinking wine while eating?

1 = do not like

2 = like slightly

3 = like moderately

4 = like very much

5 = like extremely

4. How much do you like drinking wine while not eating?

1 = do not like

2 = like slightly

3 = like moderately

4 = like very much

5 = like extremely

5. Do you have a preference for red or white wine? Yes No

If so, which do you prefer? ____
