# Supplementary figures and images for: What Can the Brain Teach Us about Winemaking? An fMRI Study of Alcohol Level Preferences
Source: PLoS One. 2015 Mar 18;10(3):e0119220. doi: 10.1371/journal.pone.0119220 (PMC4364721; doi:10.1371/journal.pone.0119220)

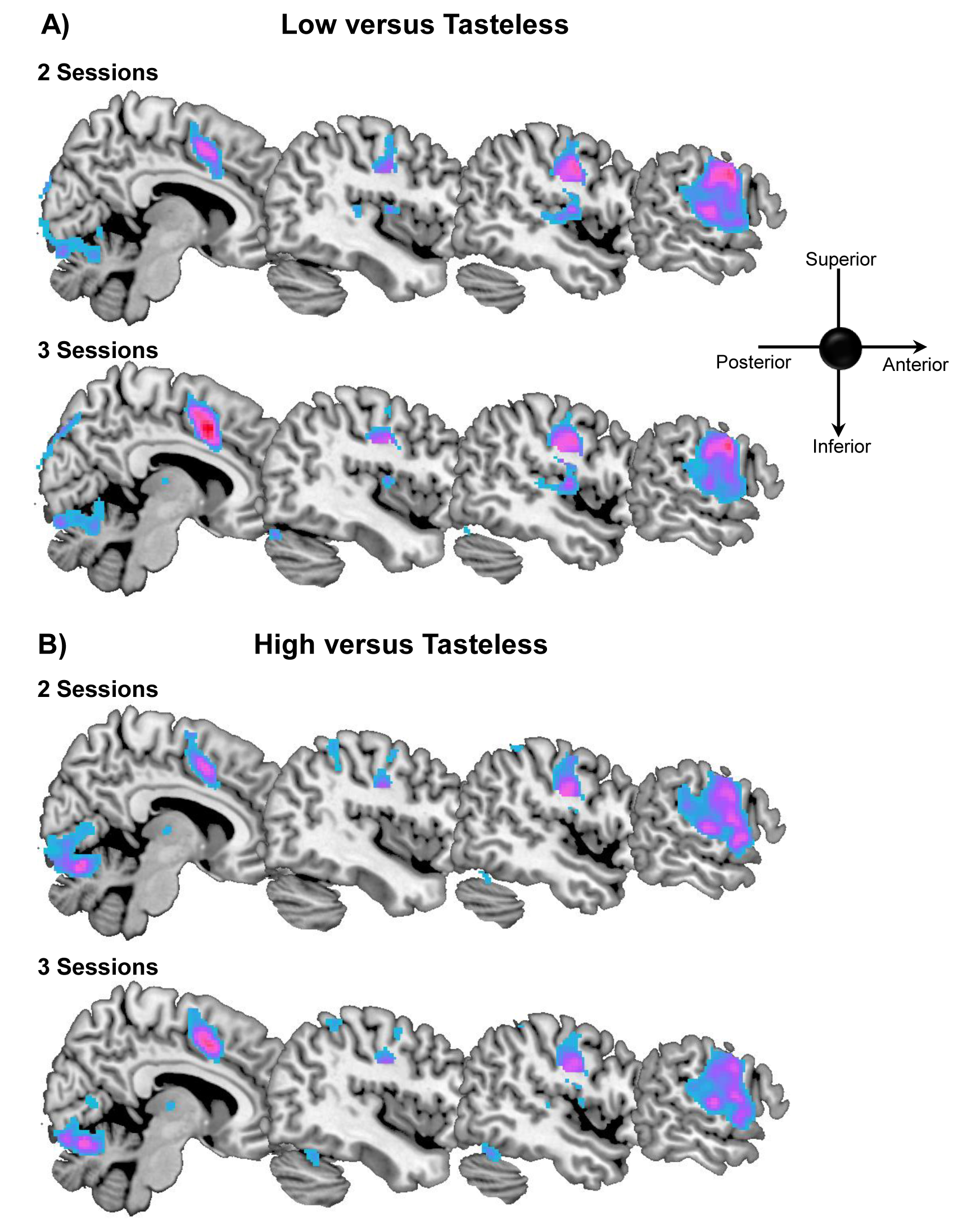

Supplement: S1 Fig — The first model with the two first sessions is represented in the upper parts A and B. The second model with the first three sessions is represented in the lower parts A and B. Note that the results are similar for 2 and 3 sessions and for the whole 4 sessions as well (See Fig. 2 and Fig. 3). (TIF) [file pone.0119220.s002.tif]
